# Mediating Role of Customer Value Co-creation and Internal Branding Between Brand Orientation and Brand Performance: Moderating Effect of Enterprise Innovative Capabilities-Evidence From Agri Product Users

**DOI:** 10.3389/fpsyg.2022.938225

**Published:** 2022-07-21

**Authors:** Zhifang Yu

**Affiliations:** Shandong College of Electronic Technology, Jinan, China

**Keywords:** enterprise brand orientation, customer value co-creation, internal branding, enterprise innovative capabilities, brand performance

## Abstract

There has been a rejuvenated interest from researchers and scholars regarding the various ways in which organizations can enhance their overall brand performance. Enterprise brand orientation are said to be the main factors that aid in improving brand performance along with internal branding and the customer value co-creation. To assess this relationship, the present study undertook an inquiry to investigate the impact of enterprise brand orientation on brand performance. Moreover, the mediating roles of customer value co-creation and internal branding were also studied. Data was obtained from 358 Chinese consumers of various household, electronic, and textile goods through a self-administered survey. A SEM technique was applied using Smart-PLS to examine the data. The findings suggested that enterprise brand orientation had a significant effect on brand performance. Moreover, it was also observed that internal branding and customer value co-creation also had a predicting role in brand performance. In addition to this, customer value co-creation and internal branding were seen to be strongly mediating the relationships between enterprise brand orientation and brand performance. The study also checked the moderating role of enterprise innovative capabilities on the effect on enterprise brand orientation and brand performance. The theoretical implication entailed the value addition made by the current study in expanding the knowledge on the predictors of enterprise brand equity. The practical implication outlined the steps that could be taken by organizations to improve brand performance through enterprise brand orientation; internal branding and customer value co-creation so that customer satisfaction and enterprise brand performance could be enhanced.

## Introduction

Branding has been considered as one of the most successful strategies of the marketing science. According to brand management studies, a good brand provides various strategic benefits such as brand expansion, recommendations, high revenues, and a better customer commitment to the corporate organizations. There has been given very little attention to assess the impact of firm branding on economic and financial performance (Seyedghorban et al., 2016). For enterprises, economic performance is vital because it is an indicator of their branding value. Therefore, firms try to evaluate the impacts of branding initiatives on their economic performance. Limited operational and financial resources of small- and medium-sized enterprises (SMEs) are the critical contributors of lower economic performance (Hirvonen et al., 2016).

Brands are considered as valuable assets of corporate sector and are helpful in proving the worth of companies. Due to the branding approach, companies become able to charge greater paybacks from their consumers. Therefore, it is not surprising that enterprises aim to establish powerful brands. A lot of research on company’s brand management is addressed from the standpoint of the consumer (Urde, 2016). Researchers have only recently begun to pay attention to product marketing from an organizational perspective. From an organizational view, the literature on brand management focuses on strategic brand management, brand orientation (BO) and internal branding (IB; Piehler et al., 2016). These three elements are considered as strong pillars of improving brand performance but hardly a connection between them has been studied so far.

The role of employees and the enterprise’s efforts to harmonize their employees with brand identities, i.e., internal branding in the enterprise’s brand strategic approach, has not been adequately examined (Tavassoli et al., 2014). Enterprises are increasingly using branding as a tactic to get a competitive advantage. In this context, Intel Corporation has now been able to earn higher amounts of money than its competitors due to its extensive branding activities (Chang et al., 2021). Therefore, recent research has urged business leaders to focus on building a BO throughout their organizations. This may occur when branding is considered as a strategic imperative. Despite the current literature’s interest in enterprise branding, our understanding is still constrained in a few areas of organizational management (Lee et al., 2019).

To prove this notion, many business owners are hesitant to adopt BO because it does not necessarily result in instant financial rewards. Business sector companies mainly focus on long-term relationship marketing but the impact of enterprise BO on long-term firm performance is somewhat understudied (Ben Youssef et al., 2018). In the context of business management, there is a need to understand that branding really has a scope in ensuring the long-term relationship marketing (Leek and Christodoulides, 2012). This is not studied before, so to fill this research gap, the current study examines the long-term effects of enterprise BO on enterprise brand performance while controlling short-term financial rewards.

There are certain concerns of buyers toward the companies like expected prices and regular interactions with manufacturers. More research is needed to properly comprehend how BO influences brand performance. It is based on the above stated concerns of customers (Guo et al., 2017). Another concern which needs to be addressed is whether all sorts of buyers have the same perception of their suppliers’ BO or not. The corporate structure of firms, particularly state ownership, is a crucial situational factor that has been overlooked by prior studies. State-owned companies tend to have more capabilities than the private ones due to set rules and regulations of operations. Research suggests that they may have different demands than private firms (An et al., 2021; Avotra et al., 2021; Yingfei et al., 2021).

Enterprise’s BO have gotten a lot of attention in organizational studies. This is partly true because they support management decisions of corporate sector. If enterprise’s BO is effectively managed then it helps organizations to achieve higher performance. Previous studies in brand management have shown that businesses with a high level of BO, outperform than those having a lower BO (Odoom, 2016). Certain external and internal conditions function as controlling factors in enterprises’ promotion of BO (Odoom and Mensah, 2019). Moreover, it was discovered that extrinsic factors impact the BO of enterprises and direct the performance of businesses (Odoom and Mensah, 2019).

Scholars have argued that the competitive aspect of corporate firms may diminish the direct performance impact of individual orientations. This thing also focused on use of interconnections between firms to create synergistic outcomes. Likewise, the research is divided as to whether enterprises should or should not explore several orientations in addition to their strengths. Research also emphasized on how an effort would improve their future performance (Laukkanen et al., 2016; Zhang et al., 2022). Brand orientation seems to be an approach to develop a company’s brand, but it can also help with other aspects of the business, such as financial success. The indirect influence of BO on economic success has been described in recent literature. As an example, researchers discovered that BO had an indirect effect on financial performance through brand performance (Urde, 2016).

A few researchers have demonstrated that corporate branding can improve economic performance through the market performance of business organizations. These findings support the theory that a company’s strategic orientation can only increase its business performance by improving its organizational efficiency (Reijonen et al., 2015). According to studies, brands assist enterprises in a variety of ways, including boosting market performance, raising quality views of their offerings, and enabling premium pricing tactics. Surprisingly, many brand managers are unaware of the strategic importance of corporate branding. Resultantly, they are unable to embrace brand strategies meaning that they do not consider enterprise branding to be a key part of their marketing plans (Urde, 2016). According to a research, business executives may be unaware of the process that connects branding initiatives to firm performance (Leek and Christodoulides, 2012). The scientific literature on enterprise BO has found that it has both positive and negative implications on firm performance (Brexendorf et al., 2015). During current times, the innovation side of businesses has been promoted as a set of complementary competencies that are great for branding. Regardless of the effectiveness of strategic competencies, literature has revealed that their complementing benefits are equivocal and not consistent between organizations (Odoom and Mensah, 2019). It is also evident from the literature that the BO of enterprises is not only directly related to brand performance, but also requires indirect ways such as IB (Chang et al., 2018; Iyer et al., 2018).

There is an assumption of IB that employees are the human capital of an organization. Their talents and expertise could be exploited to offer a sustained competitive advantage for firms. This assumption underpins the relevance of the function of IB in promoting brand performance (Iyer et al., 2018). Workers are also seen as brand advocates because their activities are critical to the successful implementation of brand strategy. Employees’ contributions to a brand’s performance are also congruent with corporate branding research. The ambition and commitment of stakeholders to carry out the brand image are critical for the establishment of a strong brand identity (Iyer et al., 2018). Human capital and employee expertise can provide essential foundation of competition in the long run. It is also evident in the case of organizational and brand performance. Internal branding is valued in management research especially, on an interpersonal basis. It lays an emphasis on the psychological perspective (Morokane et al., 2016). It is critical to understand IB from an individual perspective but it helps in assessing the influence of IB on a company’s overall effectiveness. However, there is a scarcity of research on the origins and implications of internal branding at the organizational level (Hermundsdottir and Aspelund, 2021). The current study investigates the effect of IB in supporting enterprise BO and brand performance along with a mediating effect of customer value co-creation in businesses to improve long-term brand success. Consumers are dynamic now-a-days, not just in terms of their ever-changing needs, but also in terms of their responsibilities. Their responsibilities can range from demanding product or service from companies and add in customer value co-creation (Tuan, 2017).

Consumers of different businesses have also shown the ability of value co-creation. In this context, Intel has improved its brand image as an enterprise component provider by collaborating with computer hardware makers and assemblers to create value that is visible through their products (Ballantyne and Aitken, 2007). Furthermore, the importance of customers to a company is determined by their level of attachment with the company. They regard themselves as partial members of the organizations (Tuan, 2017). They see themselves as partial members of the organization rather than as visitors. Having said that, customer-enterprise identification is defined as the degree to which consumers connect with and are dedicated to the business, which may have an impact on customers’ value co-creation behavior. It ultimately improves the brand performance (Tuan, 2017). Conversely, several gaps related to the research literature are currently visible and provide an attraction for scholastic attention and effort. To begin with, existing literature related to BO reveals a little about how its interactions with firm capabilities affect brand performance (Anees-ur-Rehman et al., 2018). Secondly, there is no empirical evidence of differences in these kinds of correlations across corporate firms in the literature. Moreover, the objective support from businesses is frequently overlooked and the BO literature appears to be scarce (Bang et al., 2016). All these gaps could have been filled by going deep into the prospects of research for brand performance. Therefore, with these objectives, current research tried to find the possible association between enterprise brand orientation with brand performance. Based on the significance and mediating roles of customer value co-creation and internal branding suggested by Chang et al. (2018) and Iyer et al. (2018), were utilized as mediators of this study. This study also evaluated the moderating effects of enterprise innovative capabilities for brand performance.

## Theoretical Basis for Hypotheses Development

The resource-based view (RBV): According to RBV, a company can obtain a competitive edge by having a match between its unique core competencies and the dynamic environment (Barney, 1991). Companies obtain splendid market positioning since each firm utilizes a distinct variety of services and competencies. The company must have assets which are valuable, unique, and incapable of being imitated or substituted (Barney, 2014). Whatever a company can update or improve is regarded as a resource. Therefore, resources might be actual or immaterial, and having access to them can provide a competitive advantage. While Barney establishes four conditions for developing a long-term competitive edge but enterprises can only fulfill a fraction of each criterion in practice (Barney, 1991).

A brand is one such resource of a company and is therefore given importance (Barney, 1991). Several academics feel that well-developed and maintained brands are significant corporate assets. Strong brands are unique, difficult to replicate, and can strengthen a firm’s performance. They can help firms establish a durable competitive edge (Fine et al., 2016). The previous study has used RBV to better understand how the brand management strategy and processes work in an organization (Baumgarth et al., 2013). The importance of IB to establish and maintain performance in the marketplace is also explained through RBV in this study. From an organizational point, the following section gives a quick survey of the literature on brand management.

Human resources, organizational learning, and intangible resources have all been emphasized as important factors in improving an organization’s performance by a number of academics (Gerhart and Feng, 2021). Based on this body of evidence, authors believe that BO is important but not sufficient for generating successful brand performance. In order to succeed, human resources (i.e., internal branding) are required. This research also gets support from upper-echelon theory (Hambrick, 2007). The theory basically provides insights about top management approaches which in this study shape the enterprise BO leading to brand performance. This theory is generally related to management, practices, characteristics and quality of services provided by the top management for brand performance. Therefore, this theory provided the foundation for enterprise BO which deals with all these aspects.

This research is also supported by the Dynamic capability theory (DCT; Teece, 2007) which lays foundations for enterprise innovation capabilities in achieving the brand performance of the enterprises. Firms develop numerous capabilities which allow them to harness and adapt existing processes and resources to accomplish business goals. Such capabilities or procedures also assist businesses in shaping their orientations to remain competitive in the face of changing market and industry situations (Odoom and Mensah, 2019). Brand orientation is also supported by this dynamic capabilities theory because of the fact that such orientation leads to improved brand performance through dynamic capabilities. Furthermore, DCT is used to explain the relationship between enterprise BOs and brand performance. In contrast to current mainstream RBV’s value appropriation/capture focus, DCT is an advancement of RBV with a specific concentration on innovation/value creation (Katkalo et al., 2010). Dynamic capabilities are defined as a company’s ability to address issues in an effective manner, as defined by its ability to detect possibilities and challenges, make effective business decisions, and modify its resource base. Growing market resources are also added with dynamic capabilities. As a result, one significant consequence of the concept of dynamic capabilities is that businesses compete not just in terms of their capacity to exploit current resources and capabilities, but also in terms of their ability to discover different resources and generate new capabilities (Teece, 2007; Tuan, 2017).

### Enterprise Brand Orientation and Brand Performance

Brand orientation is a systematic approach where the brand would become the focal point around which the organization’s processes are developed *via* stakeholder connections. As the investigations by Anees-ur-Rehman et al. (2018) show that this is intimately linked to business development and financial performance. Since the year 2000, the theoretical growth of this notion has been rising, expanding to various extensions (Sepulcri et al., 2020). Few academics developed a theoretical framework of antecedent factors of brand orientations in that sector, while Ewing and Napoli (2005) produced a scale to validate the use of nonprofit brand orientation.

Furthermore, Liu et al. (2017) discovered a positive association among brand orientation and branding mechanisms, corroborating the concept that a brand orientation strategy helps employees understand their role inside an organization. Urde (2016) coined the term brand orientation in his research study. He claimed that there have been three key forces driving companies to become more brand-oriented. Product heterogeneity is reducing, communication costs are rising, and marketplaces are becoming more integrated. Toward becoming brand-oriented, companies must spend their energy on adding value to existing brands by integrating their branding operations and making branding a greater priority on the top leadership’s agenda (Anees-ur-Rehman et al., 2018). In general, companies that establish a BO, see brands as crucial for their performance. Resultantly, corporations are prepared to invest time and money in their branding efforts. Closer relationships with customers result in higher levels of loyalty and cooperation, and higher levels of customer satisfaction. Loyalty results from greater trust and devotion and the ability to deliver more brand value leads to increased purchase intentions. Advancement of the stronger brand image and all these stated values are all advantages of BO (Chang et al., 2018). Superior brand image translates to increased brand value which influences buyers’ willingness to pay a premium price. Companies may gain from premium pricing by building a strong brand due to the price-inelastic nature of corporate buying (Ozdemir et al., 2020).

In conclusion, possessing a BO attitude enables firms to focus on ways to accomplish distinctiveness while eliminating behaviors that could devalue a business in the long run (e.g., price promotions (Wong and Merrilees, 2007). Corporations can plan and build practices that will improve long-term brand value by adopting a BO attitude. A brand-oriented attitude emphasizes the necessity of developing and maintaining a strong brand identity through time. To put it another way, BO helps to improve the internal components of corporate branding (Urde, 2016). According to prior research, possessing a BO perspective has a favorable impact on organizational or brand performance (Lee et al., 2016). All this leads to improved brand performance. Therefore, the author constructed the following hypothesis.

*H1*: Enterprise brand orientation has an effect on brand performance.

### Customer Value Co-creation

The decision-making processes of customers about purchase have been a focus of customer behavior research. It is considered that customers are more than just responders. They are also active value providers. Consumers have always been co-creators of value in the service-dominant logic. Consumers co-create value only with the firm by engaging throughout the full service-value chain as active members and cooperative collaborators in relational exchanges. Customers participate actively in the delivery of services and the fulfillment of their benefits (co-creation of value; Lee et al., 2016). Certain customers may be involved in tasks which have historically been considered organizational responsibilities like self-service, recommendations for better services, and sometimes even co-designing. Thus, customers can be characterized as part-time workers of the organization (Lee et al., 2016).

Value co-creation is accomplished in service-dominant logic through the integration of resources. Value realized from integration of resources *via* activities and interactions between contributors in the customer’s network infrastructure is how customer value co-creation is characterized. Customer participation activity and customer citizenship behavior are two higher-order variables that makeup customer value co-creation behavior. Information searching, sharing of information, appropriate behavior, and personal interaction are all examples of customer involvement behavior, which is seen as required (in-role) behavior for successful value co-creation (Lee et al., 2016). Customers are looking for guidance on how to carry out their roles as value co-creators (Yi and Gong, 2013). Consumers must also exchange information and other resources to be used in value co-creation activities. Consumers should be accountable, cooperate, follow practices and regulations, and follow guidance by staff in order to successfully co-create value with the employees of the company. Personal engagement between customers and workers is also required for effective value co-creation (Lee et al., 2016). Customer participation on the other hand, is an optional (extra-role) action that adds significant value to the organization, and it is not needed necessarily for value co-creation (Yi and Gong, 2013).

Critique, campaigning, assistance, and compassion are examples of this style of behavior. Customers supply employees with both solicited and unsolicited information, which allows employees and the business to enhance the service production process over time. Advocacy denotes a commitment to the organization and advancement of the company’s objectives over the interests of individual customers. Assisting refers to customer action that is geared at helping other customers in the setting of value co-creation. Finally, patience requires compassion and understanding on the part of the client in the event of poor service delivery that falls short of the customer’s expectations (Zhao et al., 2018).

Brand-conscious companies are more likely to recognize the importance of involving consumers in their branding strategy (Reijonen et al., 2015). The market offers in enterprise branding are frequently a mix of services and professional products. Customers are frequently encouraged to participate in the development of customized services. Whenever a company prioritizes brand (i.e., brand-orientation), it will be more ready to invest resources in creating and delivering higher brand value to its customers. Client customization and engagement are efficient methods to achieve this (Chang et al., 2018). Therefore, enterprise BO could influence customer value co-creation for the branding performance. It suggests a mediating role of customer value co-creating in improving brand performance of the companies. Companies can better understand consumer expectations and improve their marketing effectiveness/efficiency by involving customers in the value co-creation process. Furthermore, value co-creation improves shared knowledge of the brand’s worth. Consumers who actively participate in valuable co-creation initiatives are also more likely to be loyal to the partnership. Consumers are likely to provide timely feedback to suppliers when they participate in value co-creation, allowing them to promptly address possible difficulties and help improve business performance (Yi and Gong, 2013). As a result, it is expected that value co-creation will lead to improved brand performance. Based on all this supporting literature, the authors developed the following hypothesis.

*H2*: Enterprise brand orientation has an effect on customer value co-creation.

*H3*: Customer value co-creation has an effect on brand performance.

*H6*: Customer value co-creation mediates the relationship of enterprise brand orientation and brand performance.

### Internal Branding

To achieve branding goals, businesses must rely on their staff. To make sure that employees as well as the enterprise operate equally for firm’s brand values, all members of the organization must agree on the brand-building aspirations (Santos-Vijande et al., 2013). Such a strategy necessitates a firm-wide effort to guarantee that everybody including upper executives and the forward employees upholds and strengthens the company’s credibility. Such concerted effort necessitates a proper knowledge of what a brand is and what it implies for a company’s performance all across the board. As a result, businesses should educate and train all of their employees so that they fully understand and appreciate the company’s brand performance. Internal branding refers to an entire process (Anees-ur-Rehman et al., 2018).

Internal business practices through which workers grasp the branding strategy and engage with the business embody the brand. This is how internal branding is characterized. Internal branding may have been a powerful technique for aligning a company’s brand ideals with the values of its employees (Urde, 2016). Enterprises must take the initiative and develop mechanisms that allow for corporate brand value transmission. Internal branding could assist employees in effectively expressing their firm’s objectives and communicating the brand values to customers (Matanda and Ndubisi, 2013). When internal branding is done well, there is a significant level of consistency between the values of the company and the ideals of its workers. Such internal branding can boost brand performance (Anees-ur-Rehman et al., 2018). The goal of BO is to create a strong brand by maintaining brand identity during interactions between various stakeholders. Employee engagement and participation in the development and protection of the company’s brands are actively sought in brand-oriented workplace culture (M’zungu et al., 2017). The corporate branding plan is implemented through a thorough brand management approach. It includes internal branding which improves workers’ comprehension of the brand values. Resultantly, through a powerful internal branding process, employees can experience the brand when they begin to appreciate the branding concept and swear allegiance to it. The more brand-focused a company’s culture is the more successful its internal branding would be. Similarly, Zhang et al. (2016) found that in enterprise branding, BO has a significant positive link with internal branding.

Based on the foregoing discourse, internal branding is indeed an important organizational activity in the overall management of brands. It also ensures that employees are motivated to carry out the brand objectives and strategic brand management procedures to increase long-term brand equity. Internal branding is important since the brand value is co-created by customers, and impressions are formed. It happens every time when interested parties and workers engage. According to Ind (2014), brand effective and efficient management seems to be an evolving thing in which employees play a critical part in putting brand ideas into action. As a result, authors first claim that internal branding has a favorable impact on a firm’s brand performance. In addition to the link between internal branding and brand success, past research has suggested that internal branding seems to be a result of BO. For something like a brand-oriented enterprise to develop and convey brand-related objectives all across the organization, internal branding is required. Employer branding could improve employee retention, boost employee happiness, and lower-wage aspirations (Tavassoli et al., 2014). Each of these characteristics would increase motivation to strive more toward the brand’s/objectives, resulting in improved performance. Meanwhile, internal branding serves as a link here between strategic brand management strategy and brand performance by allowing an organization’s staff to concentrate on executing the brand’s long-term ambitions (Iyer et al., 2018). All this literature support leads to the following hypothesis.

*H4*: Enterprise brand orientation has an effect on internal branding.

*H5*: Internal branding has an effect on brand performance.

*H7*: Internal branding mediates the relationship of enterprise brand orientation and brand performance.

### Enterprise Innovative Capabilities

The extent to which a company’s brands, operations, and activities differ from product offerings, facilities, and technology is referred to as innovation. Several arguments and corroboration have already been offered in academia to support the idea that innovation ability is linked to company’s brand performance. Some scholars reiterate that Innovation capabilities of enterprises allows businesses to create distinctive processes and brands to acquire a competitive edge (Odoom and Mensah, 2019). Furthermore, Tajeddini et al. (2020) indicate that organizations might increase their brand competency by focusing on technological innovation and product innovation systems. Considering the paucity of data on the topic, the impact of branding and innovation complementarities on corporate performance cannot be overstated (Brexendorf et al., 2015).

According to the research, significant concentrations of innovation capabilities aid brand-oriented organizations in developing strong brands and brand assets. Additionally, branding protects, and business inventions against replication by competitors allow the firms to readily regulate risk and adapt swiftly to market developments (Brexendorf et al., 2015). Ballantyne and Aitken (2007) discovered that branding in organizations moderates the association between innovation and overall sales. From these examples, one could deduce that branding and innovation capabilities appear to act in tandem, supporting their strategic alignment.

**Figure 1:**
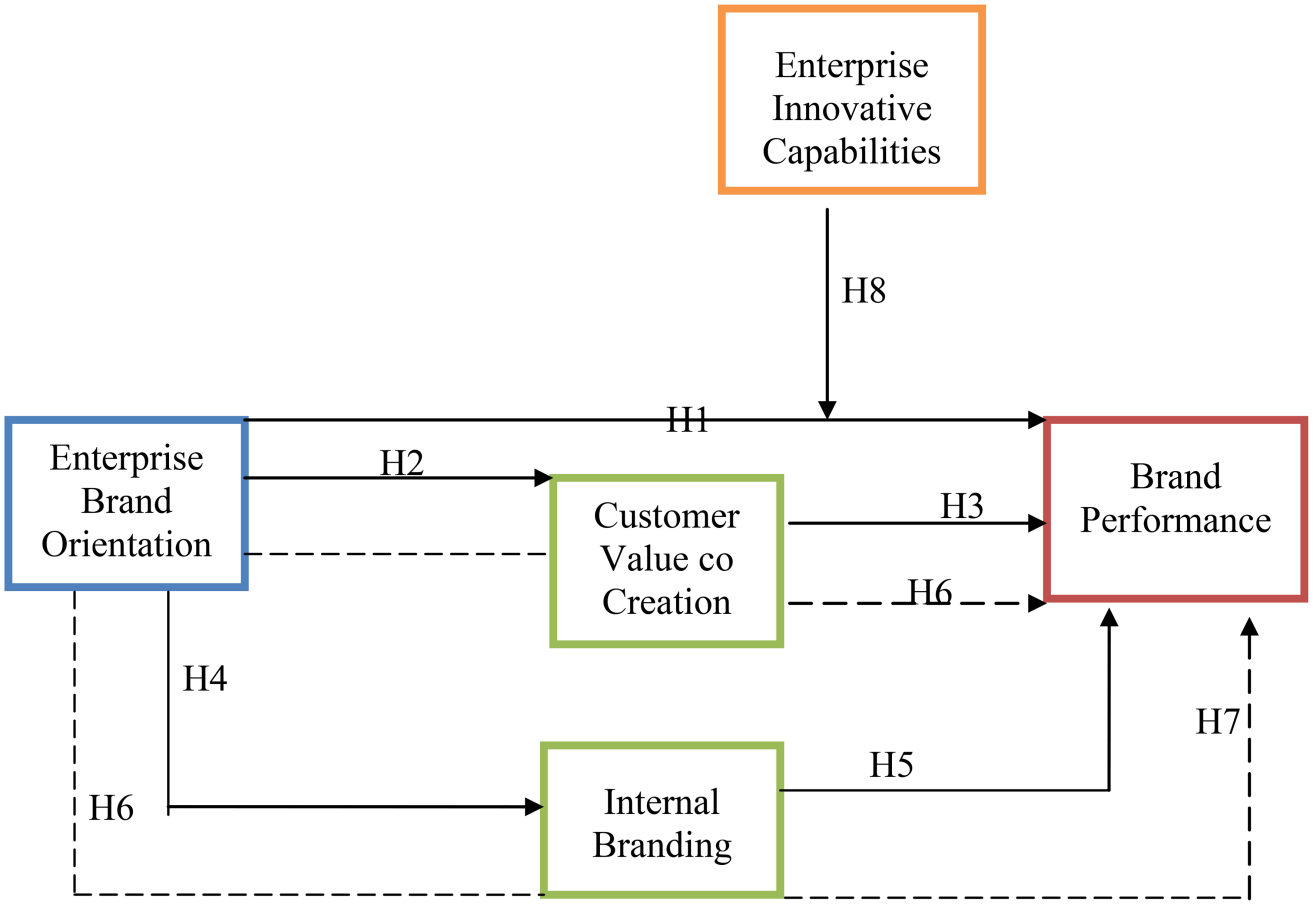
Conceptual framework.

Additionally, innovativeness emphasizes the re-invention of an organization’s processes. It also helps the development of better mechanisms by enhancing their operational adaptability. Resultantly, it is feasible to assert that process innovation affects all functional and operational areas of businesses. Innovation can also result in a significant reduction in the cost and complexity of manufacturing. This results in superior product quality, better delivery methods, a stronger brand strength, enhanced competitive advantages, and improved business performance (Maldonado-Guzmán et al., 2018). As a result of the preceding debate, the following hypothesis was established in this research. A conceptual framework is developed based on the hypothesis and literature support (see Figure 1).

*H8*: Enterprise innovative capability has a moderating effect for brand performance.

## Methodology

This chapter presents the various methods that were adopted to investigate the impact of employee efficiency and enterprise innovation on enterprise brand equity. Moreover, the mediating role of customer satisfaction was also studied. Based on the underlying objectives of the study, the research hypotheses were formulated and assessed using a deductive inquiry method. A quantitative research design was used by the researcher to eliminate the occurrence of any biases and to maintain the reliability and integrity of the data. The process of data collection was carried out with the help of survey forms that were administered to the respondents of the study. The rationality of the data was ensured by analyzing the survey form for clarity and precision. Five hundred survey forms were disbursed to the participants of the study. The population of the study was comprised of consumers who were engaged in the purchase of various household goods, electronics, and textile items. The geographical setting of the study was based in China and the unit of analysis for this study was individual.

The process of data collection was completed within 3 weeks and out of the 500 originally distributed survey forms, 358 were properly filled and returned. One hundred forty-two forms were deemed as unusable and hence discarded. Therefore, the overall response rate was 71.6%which was satisfactory as per the standards of research. The collected data was later arranged and evaluated through a specialized statistical tool. The sample size was determined using a non-probabilistic convenience sampling method. This method was selected mainly because of the fact that it significantly facilitated the researcher to obtain data within a short time and in a cost-effective manner (Scholtz and Scholtz, 2021).

### Statistical Tool

The Smart-PLS 3.3.3 software was used to examine and investigate the validity of the proposed hypotheses. Through, this software, a structural equation modeling (SEM) method was applied to determine the relationships between the constructs of the study. This tool was used because it aids the researcher to develop a path model that helps in effectively analyzing the data (Dar et al., 2022; Nawaz et al., 2022). The path models consist of the measurement and structural models. The measurement model confirms the validity of the data whereas, the structural model assesses the relationships between the constructs using t-statistics and *p*-values as key indicators.

### Measurement

The measurement scales were adopted from renowned databases and studies having a similar context. A five-point Likert scale was used to obtain responses from the participants. The scale of enterprise BO had six items and it was adopted from Anees-ur-Rehman et al. (2018). There were six items in the scale of internal branding and it was adopted from Anees-ur-Rehman et al. (2018). Furthermore, the scale of customer value co-creation consisted of four items and it was adopted from Chang et al. (2018). There were five items in the scale of brand performance and it was adopted (Chang et al., 2018). Lastly, the scale of enterprise innovative capabilities had five items and it was adopted from Odoom and Mensah (2019).

### Demographic Profile

The assessment of the various demographic traits of the participants can be viewed in Table 1. It can be seen that there were 156 males (43.5%) and 202 females (56.4%) who agreed to be a part of this study. Moreover, 58 individuals were aged between 20 to 30 years, 115 belonged to the age group of 31 to 40 years, 107 were from the 41 to 50 years age group, and 78 individuals were above 50 years of age. Furthermore, it can also be observed that 131 participants had a Bachelor’s education, 150 had a Master’s education, and 77 participants had a Ph.D. or some other qualification.

**Table 1:** Demographics analysis.

Demographics	Frequency	Percentage
**Gender**		
Male	156	43.5%
Female	202	56.4%
**Age (years)**		
20–30	58	16.2%
31–40	115	32.1%
41–50	107	29.8%
Above 50	58	16.2%
**Education**		
Bachelors	131	36.5%
Masters	150	41.8%
Ph.D. and others	77	21.5%

*N* = 319.

## Data Analysis and Results

### Measurement Model

**Figure 2:**
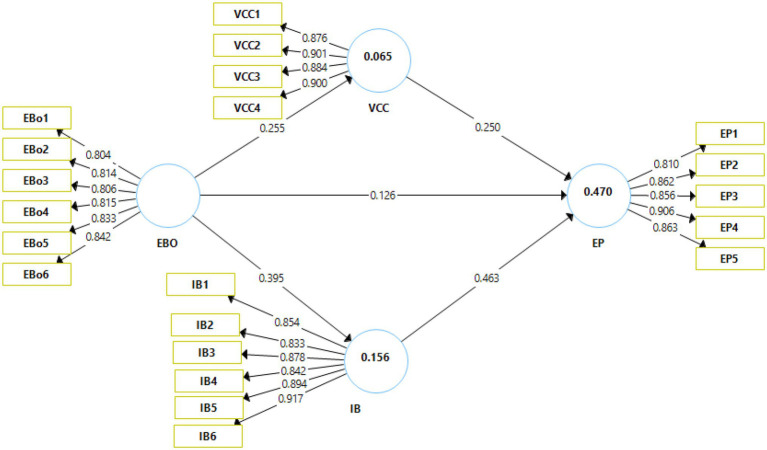
Output of measurement model. EBO, enterprise brand orientation; VCC, customer value co-creation; IB, internal branding; EP, brand performance.

The visual representation of the results of the measurement model can be seen in Figure 2. The figure denotes the relationships between the various constructs of the study.

A detailed evaluation of the outcome of the direct model can be seen in Table 2. The table denotes the values of factor loadings, VIF, Cronbach’s Alpha, composite reliability, and AVE. The factor loadings against each construct item can be seen. Bollen (2019) suggested that the acceptable values of factor loadings should be higher than 0.70. It can be seen that all factor loadings fall within the range of 0.733 and 0.913. Hence, all factor loadings can be considered acceptable. Furthermore, Hair et al. (2017) argued that the desirable VIF values should be <5. It can be observed that the highest VIF value was 3.91. Therefore, it can be deduced that all VIF values were satisfactory. Based on these observations, it can be assumed that collinearity did not exist within the data set.

**Table 2:** Model assessment (Direct model).

	Factor loadings		VIF		Construct reliability and validity		
				α	Composite reliability	AVE
Enterprise brand orientation	EBo1	0.804	2.456			
	EBo2	0.814	2.555			
	EBo3	0.806	2.442	0.902	0.925	0.671
	EBo4	0.815	2.631			
	EBo5	0.833	2.549			
	EBo6	0.842	2.591			
Brand performance	EP1	0.810	2.127			
	EP2	0.862	2.690	0.912	0.934	0.740
	EP3	0.856	2.763			
	EP4	0.906	3.590			
	EP5	0.863	2.921			
Internal branding	IB1	0.854	2.762			
	IB2	0.833	2.429			
	IB3	0.878	3.468	0.936	0.949	0.757
	IB4	0.842	2.677			
	IB5	0.894	4.421			
	IB6	0.917	4.928			
Customer value co-creation	VCC1	0.876	2.450			
	VCC2	0.901	3.798			
	VCC3	0.884	2.700	0.913	0.939	0.793
	VCC4	0.900	3.916			

The reliability of data was evaluated using the measures of Cronbach’s alpha and composite reliability. The Cronbach’s alpha values are denoted by “*α”* and they should be above 0.70 (Nawaz et al., 2021). It can be seen that enterprise brand orientation had a Cronbach alpha of 0.902, brand performance had an alpha value of 0.912, internal branding had an alpha reading of 0.936, whereas the alpha reading of customer value co-creation was 0.913. Therefore, it can be deduced that all items were internally consistent. The composite reliability was used to assess the reliability of the data set. Peterson and Kim (2013) suggested that the desirable values of composite reliability must be above 0.70. The table depicts that all values of composite reliability were above 0.70. Hence, it can be concluded that the data set was reliable. The convergent validity was assessed through the AVE values. Shrestha (2021) posited that it is desirable that the AVE values must be higher than 0.50. It was observed that all AVE values were within the range of 0.671 and 0.793. Consequently, it can be safely assumed that convergent validity existed within the data.

Table 3 given below presents the outcome of the tests that were undertaken to assess the presence of discriminant validity. These tests include the HTMT ratio and the Fornell and Larcker Criterion. Franke and Sarstedt (2019) proposed that the acceptable values of the Heterotrait-Monotrait (HTMT) ratio should be less than 0.85. It can be seen that all HTMT ratios were within the range of 0.277 and 0.689. Moreover, Fornell and Larcker (1981) argued that in the Fornell–Larcker criterion the values at the top of each column must be greater than the ones below them. This assumption was adequately met and hence it can be ascertained that discriminant validity existed within the data set.

**Table 3:** Discriminant validity.

	Fornell–Larcker criterion			Heterotrait–Monotrait ratio		
	Brand orientation	Brand performance	Internal branding	CVC	Brand orientation	Enterprise performance	Internal branding	CVC
Brand orientation	0.819							
Brand performance	0.372	0.860			0.409			
Internal branding	0.395	0.637	0.870		0.427	0.689		
CVC	0.255	0.513	0.500	0.890	0.277	0.559	0.538	

Brand orientation, enterprise brand orientation, CVC, customer value co-creation.

The sustainability of the model was studied using the values of r-square. The r-square values in proximity to 0.50 denote high model sustainability (Hair et al., 2014). The r-square values for brand performance is 0.47 and internal branding was 0.15. This indicates high model sustainability. Moreover, the inner-VIF values were also evaluated. Legate et al. (2021) proposed that the inner VIF values must be lower than 5 to eliminate the presence of collinearity. The results indicated that all inner-VIF values were well below 5. As a result, the issue of collinearity was successfully mitigated. The normed fixed index (NFI) also showed significant values. These two indicators are used to measure overall model fitness. As per Grimm and Wagner (2020) the values of SRMR and NFI should be close to 1. The results suggest that the model is highly fit for the data (NFI = 0.857).

### Structural Model

**Figure 3:**
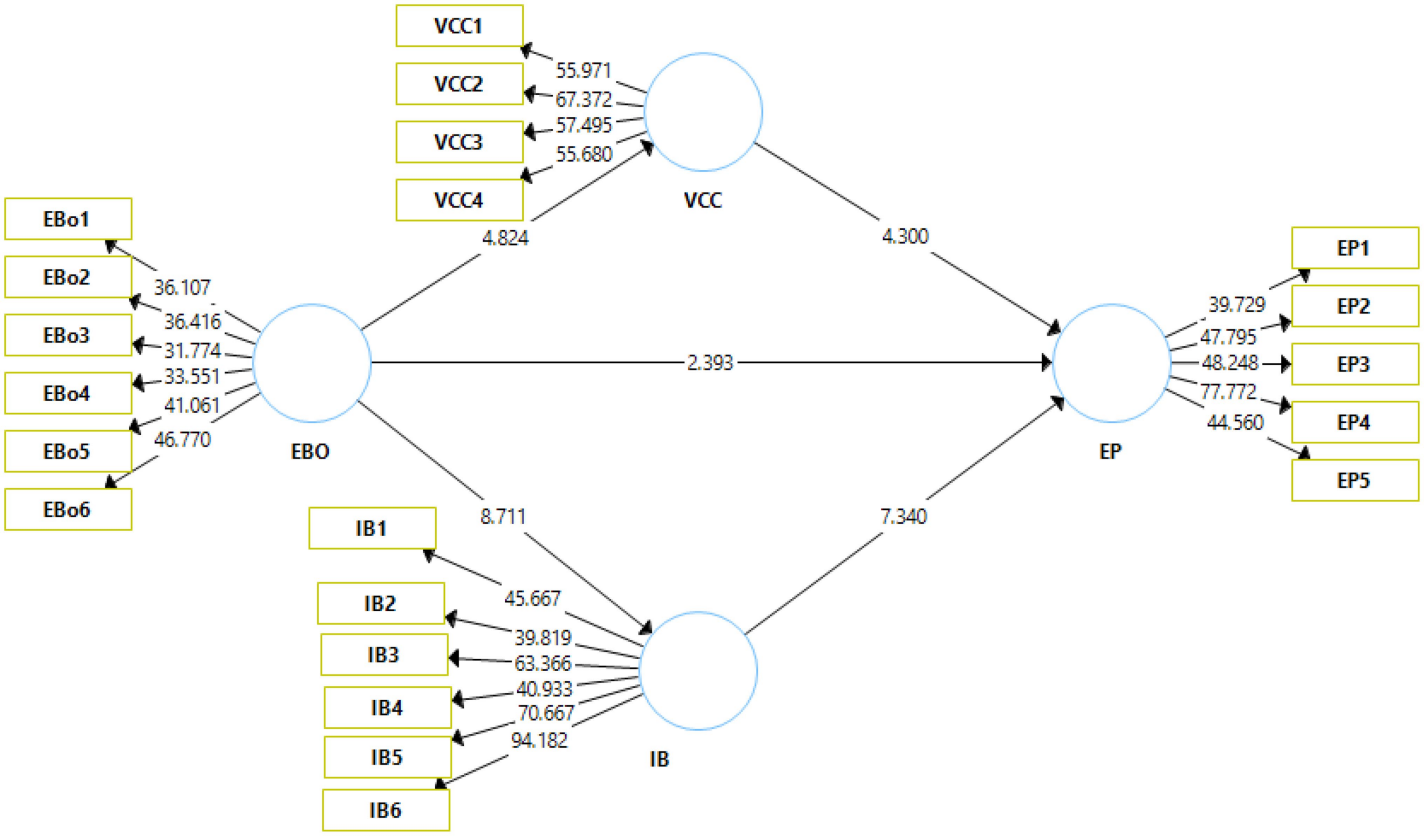
Output of structural model bootstrapping. EBO, enterprise brand orientation; VCC, customer value co-creation; IB, internal branding; EP, brand performance.

The visual depiction of the results of the structural model is shown in Figure 3. The figure depicts the structural bootstrapping method that was adopted to validate the proposed hypotheses. The bootstrapping was carried out at a 95% confidence interval.

The value of *p* and the *t*-statistic value are the key indicators that are used to make the decision on the acceptance or rejection of a particular hypothesis. Winship and Zhuo (2020) suggest that for a hypothesis to be accepted, the *t*-value should be above 1.96. On the other hand, Ioannidis (2018) posited that the value of *p* should be less than 0.05 in order to ascertain the acceptance of a hypothesis. Moreover, the effect size (f) denotes the effect of a predictor on the outcome variable (Funder and Ozer, 2019). An effect size closer to 0 is considered weak whereas, an effect size closer to 1 is considered strong.

The outcome of the analysis of the direct effects can be seen in Table 4. H1 predicted that enterprise brand orientation had an effect on brand performance. The corresponding t and *p* values are 2.393 and 0.017, respectively. Therefore, H1 has been accepted. The *f*-value (*f* = 0.025) denotes weak effect size. H2 posited that enterprise brand orientation had an effect on customer value co-creation. The results are significant (i.e., *t* = 4.824, *p* = 0.000). Hence, H2 has also been accepted. F value (*f* = 0.069) indicates weak effect size. H3 stated that customer value co-creation had an effect on brand performance. The t and value of p (*t* = 4.30, *p* = 0.000) denote that the results are significant and hence, H3 is also accepted. The effect size is weak as denoted by the *f* value (*f* = 0.088). Furthermore, H4 proposed that enterprise brand orientation had an effect on internal branding. The results are significant in nature (*t* = 8.711, *p* = 0.000). As a result, H4 is also accepted. Effect size (*f* = 0.185) is weak to moderate. H5 has been accepted that postulated that internal branding had an effect on bran performance has shown significant results (*t* = 7.340, *p* = 0.000). Effect size for this relationship is (*f* = 0.272) which shows weak to moderate effect.

**Table 4:** Direct effects of the variable.

Paths	H	O	*T*-statistics	Value of *p*	Results
Enterprise Brand Orientation → Brand Performance	H_1_	0.126	2.393	0.017	Accepted
Enterprise Brand Orientation → Value Co-Creation	H_2_	0.255	4.824	0.000	Accepted
Customer Value Co-Creation → Enterprise Brand Performance	H_3_	0.250	4.300	0.000	Accepted
Enterprise Brand Orientation → Internal Branding	H_4_	0.395	8.711	0.000	Accepted
Internal Branding → Enterprise Brand Performance	H_5_	0.463	7.340	0.000	Accepted

H, hypothesis; O, original sample.

The analysis of the indirect effects is presented in Table 5. H5 proposed that customer satisfaction (CS) mediates the relationship between employee efficiency (EE) and enterprise brand equity (EBE). H5 has been accepted as indicated by the t and value of p (*t* = 2.274, *p* = 0.023). Moreover, H6 predicted that CS mediated the relationship between enterprise innovation (EI) and EBE. The results are significant (*t* = 2.877, *p* = 0.004). Consequently, H6 has also been accepted. It can be concluded that customer satisfaction mediates the relationship between employee efficiency, enterprise innovation, and enterprise brand equity.

**Table 5:** Indirect effects of the variable.

Paths	H	O	*T*-statistics	Value of *p*	Results
Enterprise Brand Orientation → Customer Value Co-Creation → Brand Performance	H_6_	0.064	3.293	**0.001**	Accepted
Enterprise Brand Orientation → Internal Branding → Enterprise Performance	H_7_	0.183	5.805	**0.000**	Accepted

H, hypothesis; O, original sample.

**Figure 4:**
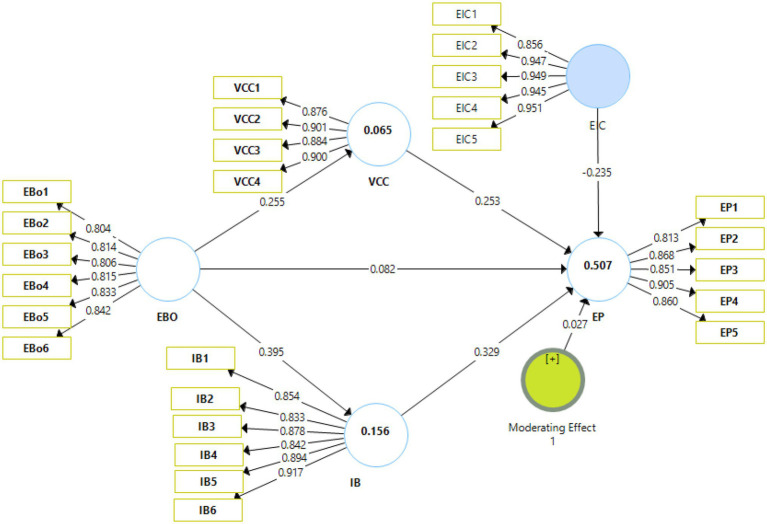
Model assessment (Moderation). EBO, enterprise brand orientation; VCC, customer value co-creation; IB, internal branding; EP, brand performance.

The factor loading for the moderating variable of enterprise innovative capabilities is also found to have significant factor loading for all the items (i.e., above 0.7). Further, the Cronbach alpha and the composite reliabilities also found to be above 0.7 showing that internal consistency of the variable. In addition, the average variance extracted was found above 0.765 which shows that variance is explained more than the error hence meeting the criteria. Therefore, Table 6 shows the model assessment with moderation which is acceptable. Similarly, the HTMT ratios and Fornell and Larcker criteria obtained with the new variable of enterprise innovative capabilities also showed significant results (Figure 4).

**Table 6:** Model assessment (Moderation).

	Factor loadings		Construct reliability and validity		
			α	Composite reliability	AVE
Enterprise brand orientation	EBo1	0.80			
	EBo2	0.81			
	EBo3	0.80	0.902	0.925	0.671
	EBo4	0.81			
	EBo5	0.83			
	EBo6	0.84			
Brand performance	EP1	0.81			
	EP2	0.86	0.912	0.934	0.740
	EP3	0.85			
	EP4	0.90			
	EP5	0.86			
Internal branding	IB1	0.85			
	IB2	0.83			
	IB3	0.87	0.936	0.949	0.757
	IB4	0.84			
	IB5	0.89			
	IB6	0.91			
Customer value co-creation	VCC1	0.87			
	VCC2	0.90			
	VCC3	0.88	0.913	0.939	0.793
	VCC4	0.90			
Enterprise innovative capabilities	EIC1	0.85			
	EIC2	0.93			
	EIC3	0.84	0.901	0.920	0.765
	EIC4	0.91			
	EIC5	0.85			

**Figure 5:**
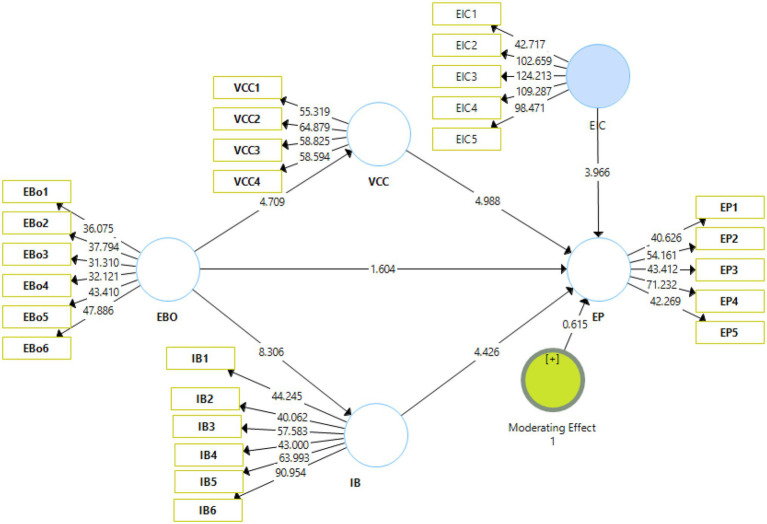
Structural model bootstrapping (Moderation). EBO, enterprise brand orientation; VCC, customer value co-creation; IB, internal branding; EP, brand performance.

The analysis of the moderating effects is presented in Table 7. H8 proposed that enterprise innovative capabilities do not moderate the effect of enterprise brand orientation on brand performance. H8 has been rejected as indicated by the *t* and value of *p* (*t* = 0.615, *p* = 0.539) (Figure 5).

**Table 7:** Moderating effect.

Paths	H	O	*T*-statistics	Value of *p*	Results
EIc_Mod → Brand Performance	H_8_	0.027	0.615	0.539	Rejected

EIC_Mod, Enterprise innovative capabilities.

## Discussion

The current research focused on mediating roles of customer value co-creation and internal branding between enterprise brand orientations and brand performance. Variations in BO and brand performance relationships are more or less significant for firms. It explores the moderating elements of a company’s innovative capabilities (Odoom and Mensah, 2019). The research postulated positive benefits of innovation as complementing enterprise capabilities which enhance the BO and brand performance relationship among firms (Baumgarth et al., 2013). The study’s findings are supported by earlier research in a variety of ways. Overall, our research demonstrates a favorable association between BO and brand performance, as well as a substantial positive relationship within the parameters of customer value co-creation and internal branding. This research was conducted to find out the possible positive and significant association between enterprise BO and brand performance. As it was previously elaborated in many investigations that BOs had been significantly associated with brand performance whether it be financial or relational (Anees-ur-Rehman et al., 2018; Chang et al., 2021). This research also found similar results indicating that enterprise BOs were positively and significantly connected with the brand performance of the enterprises.

This is due to the fact that top management shapes the orientations of the enterprises keeping in view the ultimate outcome of the decisions and strategies. Therefore, these results were obtained showing a positive and strong association. In the current investigation, some other direct associations were also evaluated. The direct association between enterprise BO with customer value co-creation produced a significant association. The possible reason behind such results lies in the fact that strategic planning of the enterprises is done keeping in mind the importance of the customers. The customers share the values of the brands and act as a stakeholder in brand promotion (Reijonen et al., 2015). Therefore, BOs are directly linked with customer value co-creation. The impact of customer value co-creation showed a strong association with the brand performance which again showed that shared value between customers and the associated enterprise work on the shared objectives. Therefore, customer value co-creation leads to improved brand performance. Similar kinds of results have been previously published by many scholars such as Yi and Gong (2013). The values of the enterprises and the customers had shown a strong mediating impact between enterprise BO and brand performance. These kinds of mediating effects of customer value co-creation indicated that direct effects of enterprise BO and brand performance could be more enhanced if customers are also kept in focus. This could lead to improved brand performance for the employees.

These kinds of results were also reported by Chang et al. (2018) previously, which affirmed the significance of the customer value co-creation in the context of enterprise brand performance. This study also looked into the possible relationship between enterprise BOs and internal branding. The results are supported by the many researchers in the recent past indicating that BOs are directly and significantly related to internal branding. As an internal branding approach, employees of the enterprises are kept in the direct focus of the organizations. Similarly, like the customers, employees are also the stakeholders who work for the branding of the enterprises (Eid et al., 2019). These results are also in accordance with some previous researchers who affirmed that there was a significant association between enterprise BO and internal branding of the enterprises (Anees-ur-Rehman et al., 2018). The results also indicated that internal branding affected the brand performance positively and also mediated the relationship of enterprise brand performance. The results were also in accordance with some of the previous research which looked into the direct relationships between internal branding with brand performance (Iyer et al., 2018). This proved that proper internal branding of the employees could lead to the improved brand performance of the enterprises. The mediating role of internal branding was also significant between the relationship of enterprise BO and the brand performance of the enterprises. This role of internal branding boosted the relationship between both.

It indicated that spending resources on the employees, enhance the performance of the brands positively. Previously, the mediating role of internal branding was also evaluated in different contexts showing a strong role of it (Iyer et al., 2018). Moreover, the moderating effects of enterprise innovative capabilities could not influence the brand performance in this research. This is possibly due to the fact that BOs were strongly associated with brand performance. Therefore, enterprise innovative capabilities could not show its regulating role, or these were also part of the branding of firms. Although, some researchers indicated that enterprise innovative capability regulated the BO and brand performance. It might happen due to a lack of mediators between both in the previous study (Maldonado-Guzmán et al., 2018).

### Theoretical Implications

There are some key and valuable theoretical implications associated with this study. This research significantly adds to the RBV of firms. The theory focuses on the improvement of resources for the betterment in performance. While, this research is a significant contribution in the management sciences. The resources of companies are enhanced by incorporation of internal branding strategies. It leads to the development of a brand. Therefore, brand performance of such companies is improved. This adds to the underpinned theory of RBV. Moreover, this research also backs the underpinnings of DCT which is focused on improving innovative capabilities of firms. Improved innovative capabilities add in to brand performance. In this way, both of these theories get a strong support from current research. It also signifies the importance of current research in which impacts of BO on brand performance are evaluated.

Brand performance is significantly improved through integrated approach of BO, innovative capabilities, and customer value co-creation. Firstly, this study undertook a rigorous inquiry to produce empirical evidence that indicates the presence of a strong relationship between enterprise brand orientation, internal branding, customer value co-creation and the brand performance. By undertaking this inquiry, this study addressed the scarcity of knowledge that was present on this subject. It was established that by making considerable investments in enhancing brand orientation of the organization and deploying internal branding in the business processes, an organization can significantly enhance its overall brand performance. Secondly, the present study provided strong theoretical evidence by assessing the mediating role of customer value co-creation and internal branding in enhancing the relationship between enterprise brand orientation and brand performance. Both of these inquiries have significantly added value to the existing literature that is available on the predictors and antecedents of enterprise brand orientation, internal branding and the brand performance.

### Practical Implications

The results of this study present some key practical implications for managers and businesses. (1) The results suggest that the organizations should make considerable efforts toward enhancing the overall enterprise brand orientation that counts for the improved brand performance. This can be achieved by setting realistic work objectives and by effectively communicating work expectations to the employees that would contribute to creating value for the customers along with employees. (2) Moreover, the employees need to be provided with a adequate motivation and productive work environment so that they can maximize their performance on the job and contribute to enhancing the brand performance. (3) Further, the businesses should also make considerable investments in not only creating but adopting and implementing such practices to provide smooth and reliable services to the customers that would add to the branding of the organization. (4) Furthermore, the organizations should also develop a customer satisfaction blueprint and train its workforce to follow customer satisfaction protocols in order to maximize customer value co-creation to enhance the customer base. These efforts would in turn cause an enhancement in the overall brand performance.

### Limitations and Directions for Future Research

The limitations that were associated with this study included a restricted sample size. (1) The sample size could be enhanced by future studies to yield more generalizable results. The present study was undertaken within the geographical location of China. This study should be conducted in other cultural contexts and regions to improve the generalizability of the findings. (2) This study adopted a cross-sectional design. Therefore, future studies should use a longitudinal design to acquire data from respondents at multiple points in time. This would significantly improve the reliability of the results. (3) Lastly, future studies should incorporate other mediating variables like customer satisfaction and measure the brand performance through employee efficiency and enterprise innovation. Future research should introduce other potential moderators such as perceived brand image and role clarity in order to broaden the understanding of the factors that influence enterprise performance.

## Conclusion

Brand performance has recently received renewed interest from researchers and academic scholars. Previously, brand performance was mainly studied from the perspective of the organizations and there was a dearth of knowledge regarding the factors customer value co-creation. Therefore, this study undertook a rigorous inquiry to determine the impact of enterprise brand orientation on brand performance. This was done in the presence of customer value co-creation and internal branding as mediators. It was concluded that both enterprise brand orientation in the presence of internal branding customer value co-creation had a significant effect on brand performance. It was also noted that internal branding and customer value co-creation innovation had an effect on brand performance. Moreover, internal branding and customer value co-creation significantly mediated the relationship between employee efficiency, enterprise innovation, and enterprise brand equity. However, the moderating role of enterprise innovative capabilities could not significantly impact the relationship of enterprise brand orientation and brand performance.

## Data Availability Statement

The original contributions presented in the study are included in the article/supplementary material, further inquiries can be directed to the corresponding author.

## Ethics Statement

The studies involving human participants were reviewed and approved by Qilu Normal University, China. The patients/participants provided their written informed consent to participate in this study. The study was conducted in accordance with the Declaration of Helsinki.

## Author Contributions

ZY conceived, designed, and wrote the paper. The author read and agreed to the published version of the manuscript.

## Funding

The Subject of the “14th Five Year Plan” of Educational Science in Shandong Province in 2021 (Research on the Ability Improvement of New Professional Farmers Supported by E-commerce Majors in Higher Vocational Colleges 2021YB062).

## Conflict of Interest

The author declares that the research was conducted in the absence of any commercial or financial relationships that could be construed as a potential conflict of interest.

## Publisher’s Note

All claims expressed in this article are solely those of the authors and do not necessarily represent those of their affiliated organizations, or those of the publisher, the editors and the reviewers. Any product that may be evaluated in this article, or claim that may be made by its manufacturer, is not guaranteed or endorsed by the publisher.
